# Considerations of valvular heart disease in children with ventricular assist devices

**DOI:** 10.3389/fcvm.2023.1056663

**Published:** 2023-03-22

**Authors:** Ming-Sing Si, Vikram Sood, Reshma Biniwale, David Peng

**Affiliations:** ^1^ Department of Pediatrics, Division of Pediatric Cardiology, University of Michigan, C.S. Mott Children’s Hospital, Ann Arbor, MI, United States; ^2^ Department of Cardiac Surgery, C.S. Mott Children's Hospital, University of Michigan, Ann Arbor, MI, United States; ^3^ Department of Surgery, Division of Cardiac Surgery, University of California, Los Angeles, Mattel Children’s Hospital, Los Angeles, CA, United States

**Keywords:** pediatric, heart failure, ventricular assist device, valvular heart disease, valve surgery (or cardiac surgery)

## Abstract

Ventricular assist devices have become a valuable tool in the treatment of heart failure in children. The use of ventricular assist devices has decreased mortality in children with end-stage heart failure awaiting transplant. It is not uncommon for children with end-stage heart failure associated with cardiomyopathy or congenital heart disease to have significant systemic semilunar and atrioventricular valve regurgitation, which can impact the efficiency and efficacy of hemodynamic support provided by a ventricular assist device. Therefore, implanting clinicians should carefully assess for valve abnormalities that may need repair and impact device selection and cannulation strategy to effectively support this diverse population. The purpose of this review is to provide an overview of this important and relevant topic and to discuss strategies for managing these patients.

## 1. Introduction

The treatment of heart failure in children and adults has been revolutionized by the advent of ventricular assist devices (VADs) which can reliably restore normal levels of cardiac output. VADs that are used for long-term support in children, which in the United States currently include the EXCOR Pediatric device (Berlin Heart GmbH, Berlin, Germany) and the HeartMate 3 device (Abbott Cardiovascular, Plymouth, MN, USA), can serve as a bridge to heart transplant or, in rare cases, as a bridge to recovery in children with end-stage heart failure. The majority of children undergoing heart transplantation either have cardiomyopathy or end-stage congenital heart disease (CHD) that results in severe systolic and/or diastolic ventricular dysfunction and over the past two decades, the presence of VAD support in children eventually undergoing heart transplantation has doubled (1). Although the prevalence of valvular heart disease in children undergoing VAD implantation and support has not been specifically defined, it is not uncommon for patients with cardiomyopathy or CHD to also have concomitant valvular heart disease (2, 3). In some patients, valvular heart disease may be the result of a poorly functioning ventricle and in other instances, a chronically malfunctioning heart valve may be the cause of ventricular dysfunction (4, 5). Regardless of the etiology, the presence of valvular heart disease can impact the efficacy of VAD support and thus may require surgical intervention at the time of or after VAD implantation. In this review, we will discuss the extent, impact, diagnosis, and treatment of valvular heart disease in children with end-stage heart failure who are being considered for VAD therapy. Much of the discussion presented here is based on evidence from studies in adult VAD patients, however, the pathophysiological considerations of valvular heart disease are similar and thus relevant to pediatric patients.

## 2. Pathophysiology

The affected heart valve, type of valve lesion (i.e., stenosis or regurgitation), type of VAD, and VAD cannulation sites influence the impact of valvular heart disease on the efficiency of VAD support. In the following discussion of each abovementioned factors, we will use the scenario of a VAD supporting the systemic circulation in a biventricular heart. This discussion can be extended to the scenario of a VAD-supported pulmonary circulation as well as a VAD-supported single ventricle heart.

**Figure 1:**
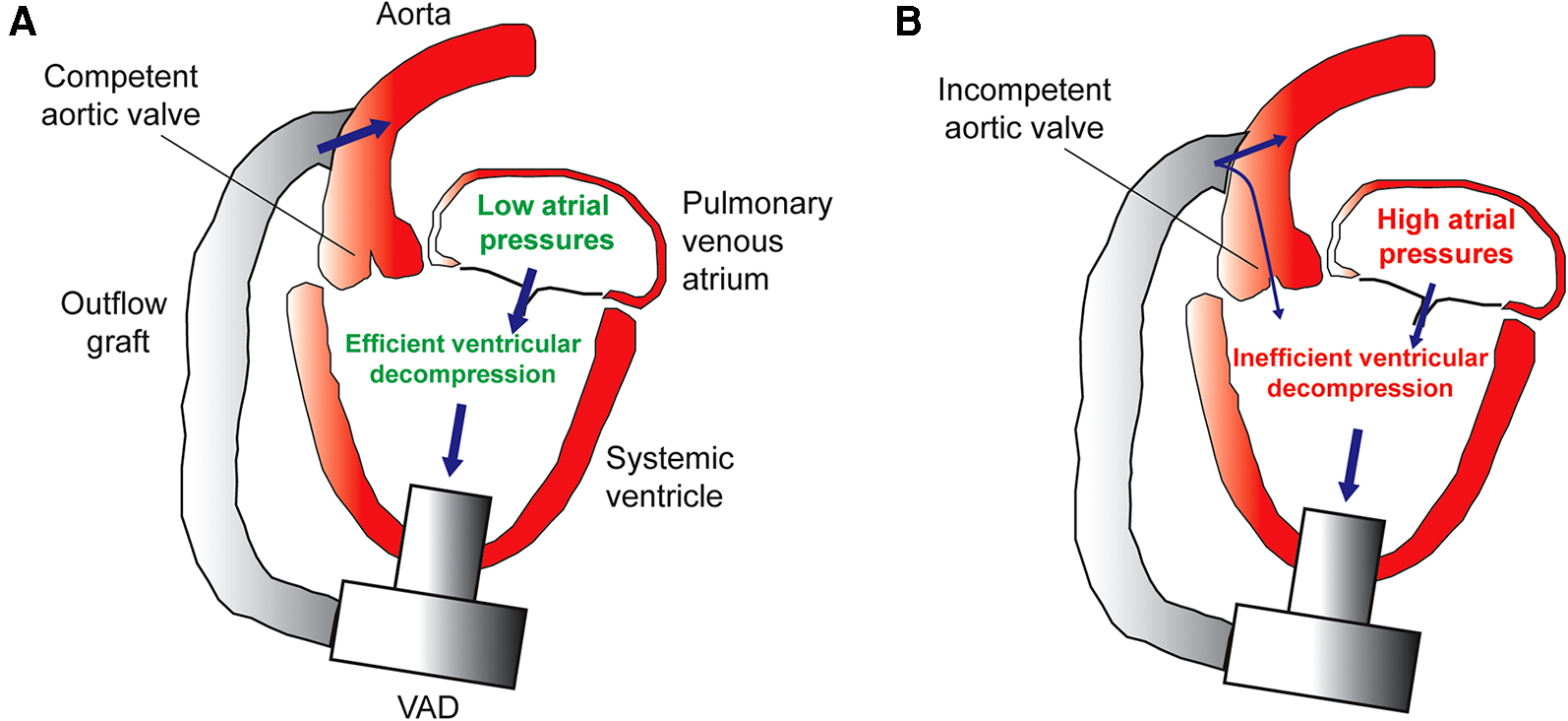
(**A**) Efficient decompression of the systemic ventricle and pulmonary venous atrium in the setting of a competent aortic valve. (**B**) Inefficient VAD support caused by aortic insufficiency leads to incomplete ventricle decompression, high atrial pressures, pulmonary venous congestion, and heart failure symptoms.

In general, all VADs have an inflow and outflow and there should be at least one competent valve somewhere between the VAD inflow position and the aorta (VAD outflow position). In the most common situation of the VAD inflow cannula placed in the left ventricle and the VAD outflow connected to the aorta, aortic valve insufficiency (AI) would lead to the recirculation of VAD flow and thus loss of systemic cardiac output (6, 7) (Figure 1). The extra regurgitant volume may also lead to increased mitral regurgitation (MR), less emptying and decompression of the left atrium and pulmonary venous bed, increased pulmonary artery pressure and right ventricle workload, right ventricular failure, and congestive heart failure symptoms. Aortic insufficiency in left ventricle assist device (LVAD) patients can lead to an increase in left ventricle end-diastolic pressures, which has been associated with poorer clinical outcomes (8). Aortic valve insufficiency in adult VAD patients develops over time and has been associated with poor clinical outcomes including increased mortality (9, 10). Also, mild AI that is present at the time of VAD implantation in adult patients can worsen with time (10–13). The tendency of the aortic valve to leak or become more regurgitant after VAD implantation (especially continuous flow VADs) is likely secondary to ultrastructural and degenerative changes in the leaflets (14, 15). The expression of genes encoding the inflammatory cytokines interferon gamma, interleukin 1 beta, and tumor necrosis alpha have been found to be increased in the aortic valve leaflet tissue of VAD patients (16). Furthermore, the angle at which the VAD outflow graft is anastomosed to the aorta has been found to influence the development of AI in adult VAD patients (17) and the same investigators have confirmed this finding in a large animal model (18). The increased transvalvular gradient present in both systole and diastole in the VAD-supported systemic circulation has also been postulated to contribute to aortic valve leaflet degeneration and insufficiency (19). A computational fluid dynamics study revealed that adult VAD patients who developed *de novo* AI after VAD implantation have higher localized wall shear stress on the aortic valve leaflet tips as compared to VAD patients who did not develop AI (20).

Since the AI is often continuous with continuous-flow VADs, even “mild to moderate” continuous AI can be deleterious (21). Because AI has been determined to be a time-dependent phenomenon in adult patients, we surmise that in pediatric patients the development of new AI may be less important since the duration of VAD support tends to be shorter in children (22). However, with longer-term therapy and the increasing number of destination devices in recent years (23), we can expect to see more AI in these chronically-supported children.

In the scenario of the VAD inflow cannula placed in the left atrium, mitral valve insufficiency (MR) may be well tolerated *as long as* the aortic valve is competent. The same cannulation strategy can also be successful in the setting of heart failure with preserved systolic function, a competent mitral valve, and a mildly insufficient aortic valve. In this scenario, VAD recirculation would be prevented by the competent mitral valve and the LV with normal systolic function should be able to eject the regurgitant volume from the mildly insufficient aortic valve. Certainly, this arrangement would not be tolerated if the left ventricular systolic function was decreased as left ventricular dilation would result in subsequent MR and impact right ventricle function.

Mitral valve stenosis can limit the efficiency of VAD output if the inflow cannula is positioned in the left ventricle and significant gradients (mean > 10 mmHg) have been recommended as a reason to intervene on the mitral valve in adult patients (7). If the VAD inflow is positioned in the left atrium, then mitral stenosis should have no effect on VAD support. Aortic stenosis without AI is usually well tolerated and should not affect VAD support efficacy.

The type of VAD can also influence the clinical impact of mitral valve regurgitation. The EXCOR Pediatric VAD is a paracorporeal, pulsatile device and is used in infants and small children (24, 25). The HeartMate 3 VAD is an implantable, continuous flow device that can be utilized in larger children and adolescents with heart failure (26). Mitral insufficiency may be tolerated with a continuous flow VAD with the inflow positioned in the left ventricle as long as the ventricle is effectively offloaded by the VAD throughout all stages of the cardiac cycle, and thus the ventricular dilatation and regurgitant volume into the left atrium may be decreased. In a small pediatric patient with mitral regurgitation and the same cannulation strategy, a pulsatile flow VAD may not be as effective in decompressing the ventricle during diastole and thus mitral regurgitation may be more important. The inefficient unloading of the left ventricle during systole may even be more significant if a large size Berlin pump is utilized with a slower pump rate. Therefore, in small children with significant MR who are being considered for an Excor Pediatric VAD with the inflow positioned in the left ventricle, intervention for the mitral valve should be considered.

## 3. Epidemiology

There is a paucity of data on valve dysfunction and concomitant valve procedures performed at the time of VAD implant in pediatrics. One series of 45 patients with Fontan circulation supported with VAD reported severe atrioventricular valve regurgitation in 43%, mild aortic insufficiency in 28%, and moderate aortic insufficiency in 8% of patients (27). The precise prevalence of valvular heart disease in children undergoing VAD implantation is not known.

## 4. Diagnosis of valvular heart disease in children undergoing VAD implantation

Diagnosis of valvular heart disease in pediatric patients considered for VAD support or after the initiation of VAD support is usually diagnosed by transthoracic echocardiography (28). Transesophageal echocardiography in the operating room can further define the presence, extent, and mechanism of valvular disease prior to VAD implantation (28). Importantly, a transesophageal echocardiogram is utilized to monitor the presence of existing or the development of new AI immediately after VAD implantation. Intraoperatively, an epicardial echocardiogram (29, 30) may provide alternative views of the semilunar or atrioventricular valves if the transthoracic or transesophageal approaches do not provide a complete assessment of the valves. Cardiac magnetic resonance, usually obtained to assess ventricular function, fibrosis, and cardiac anatomy, can be particularly useful for quantifying the degree of valvular regurgitation or stenosis prior to VAD implantation (31, 32).

In patients who develop *de novo* or progressive AI after VAD implantation because of the increased transvalvular gradient caused by enhanced pressurization of the aorta (33), the combination of echocardiography and cardiac catheterization during a ramp trial may be informative as to the degree of ventricular congestion and low systemic cardiac output caused by the AI (8, 34). Furthermore, it has been proposed that aortic valve regurgitant volume can be calculated by multiplying the proximal isovelocity surface area by the aortic regurgitant time (both echocardiography-determined parameters), although this approach needs further validation (35).

## 5. Management of valvular heart disease at the time of VAD implantation

In general, surgical intervention for regurgitant heart valves are either repair or replacement. A multitude of techniques have been developed for mitral valve repair and several options exist for mitral valve replacement in older children (36–38). Techniques for aortic valve repair in children are not as developed, especially in smaller patients. Concomitant surgical valve repair or replacement in the setting of VAD implantation setting should be expedient and lead to durable resolution of the valve lesion. With these goals in mind, in the following section, we provide discussion of relevant surgical interventions for valvular heart disease in children undergoing VAD implantation.

### 5.1. Mitral valve disease

There is a dearth of literature describing mitral valve procedures in pediatric patients undergoing VAD implantation. The adult-focused literature, on the other hand, has demonstrated that significant, functional MR is common in patients undergoing VAD implantation (7, 39, 40), and that MR usually improves with continuous flow VAD support (41). Interestingly, though, there are recent, adult-focused data that suggest that there are a subset of patients undergoing continuous flow VAD implantation, with severe MR, who benefit from concomitant mitral valve repair (42–44).

On the other hand, in children being considered for pulsatile flow VAD implantation, those with moderate or greater regurgitation should undergo mitral valve repair. In children with hypertrophic or restrictive cardiomyopathy and with a continuous flow device, mitral valve replacement may not be appropriate for the smaller hearts, for whom an atrial location for the inflow cannula or complete excision of the obstructing systemic atrioventricular valve is indicated. While complex and elegant repair techniques have been developed for regurgitant mitral valves (38), these often are not indicated during VAD implantation because of the additional cardiopulmonary bypass and cross-clamp times needed to execute them. The most common mitral valve procedures at the time of VAD implantation include mitral valve repair: edge-to-edge approximation (45) or partial or complete annuloplasties (46). Finally, in the patient in need of concomitant mitral surgery—in whom repair is not felt to be feasible—mitral replacement with a bioprosthesis should be considered.

The edge-to-edge technique of mitral valve repair was introduced in the late 1990s by Alfieri and colleagues (47). The original technique was first established for complex repair of degenerative MR in adults, however, its use has expanded over the past twenty years to other modalities of MR (Barlow's, functional, etc.). In patients undergoing VAD implantation, edge-to-edge repair is performed transapically, while on cardiopulmonary bypass, but without arresting the heart (44, 45). After the left ventricle apex has been cored and excised, blood in the left ventricle and left atrium are suctioned with a cardiotomy suction to aid in visualization of the mitral valve. The anterior and posterior leaflets of the mitral valve are fixed to one another at the A2/P2 position with a mattress-fashioned, Polypropylene suture, which is tied down over a felt pledget. Importantly, this suture is placed and tied down on the *ventricular* side of the mitral valve apparatus. The remainder of the VAD procedure is undertaken. The edge-to-edge technique adds little cardiopulmonary bypass time and does not require a separate access incision to visualize the mitral valve. It is, however, a view of the mitral valve that surgeons are not particularly familiar with, which leads many surgeons to be uncomfortable about employing this technique.

Annuloplasty repair techniques for mitral valve repair are popular as adjuncts to complex mitral valve repair in the setting of degenerative MR in adults. In the setting of VAD implantation, annuloplasty is typically performed *via* a trans-septal incision, while on cardiopulmonary bypass, and without arresting the heart (46). Unlike in repair of degenerative MR, functional MR in the adult undergoing VAD implantation is typically repaired with a complete ring. Pediatric patients may undergo limited or partial annuloplasty, as ring sizes are often too large for this patient population. In concomitant mitral valve repair with an annuloplasty, the left ventricle apex is cored to fully decompress the ventricle and prevent air embolism, the atrial septum is incised in a longitudinal fashion, and a self-retaining retractor is placed to expose the mitral valve. Horizontal mattress sutures are placed, circumferentially, in the mitral valve annulus. An annuloplasty ring is sized and selected, the sutures are placed through the ring, the ring is seated, and the sutures are tied down. In a pediatric patient that is not undergoing ring placement, a limited or partial annuloplasty is performed. The atrial septum is closed and the remainder of the VAD procedure is undertaken. Notably, the annuloplasty technique of repair does add a significant amount of time to the VAD procedure, however it provides access to the mitral valve in the surgeon's conventional view.

### 5.2. Aortic valve disease

Multiple single center studies have demonstrated that AI post LVAD implantation in adult patients can lead to poor clinical outcomes and ongoing heart failure (10, 48, 49). However, a recent analysis of the ISHLT Mechanically Assisted Circulatory Support (IMACS) Registry spanning the years of 2013–2017 demonstrated several notable findings (50). First, survival was not significantly different in VAD patients with moderate to severe AI who underwent aortic valve intervention (replacement or repair) as compared to patients who did not receive any intervention (50). Second, when considering all patients who underwent aortic valve procedures (i.e., those with all grades of AI), survival was observed to be inferior to patients who did not receive aortic intervention (50), suggesting that those with no or only mild AI had especially worse survival who underwent aortic valve surgery than those who did not undergo aortic valve surgery. These results are difficult to interpret, given that the recurrence of AI after aortic valve surgery was not noted and is an important limitation of this registry study. Furthermore, the degree of variability in the assessment of the severity of AI and heterogeneous criteria for the indications used by caregivers to proceed with aortic valve surgery are other important confounding factors not accounted for in this study. Nonetheless, we believe that ongoing and significant heart failure symptoms in the setting of systemic VAD support and at least moderate AI are indications for aortic valve intervention. The appearance and persistence of new, moderate AI just after systemic VAD implantation in children despite decreasing pump speed to the lowest, tolerable speed also warrants careful consideration of immediate aortic valve intervention in the operating room, as described in the following paragraphs.

The insufficient aortic valve in the pediatric patient undergoing VAD implantation can be repaired with a central coaptation stitch (51). In this method, which was originally described in adults, left ventricular ejection will still allow for opening of the aortic valve, though the effective orifice is reduced. During VAD implantation, the heart is arrested, and the ascending aorta is incised on its rightward surface—in the location of the eventual outflow graft anastomosis site. Via the aortotomy, the aortic valve is inspected to determine the mechanism of AI. If no other repair is deemed appropriate, a pledget-supported polypropylene suture is used to approximate the three nodules of Arantius (51). The outflow graft is then sewn to the aortotomy site in an end-to-side fashion, and the remainder of the VAD procedure is completed. The central coaptation stitch is feasible and has been utilized in pediatric patients. Aortic valve repair by placement of a subannular annuloplasty ring along with leaflet free-edge plication has been described in an adult patient undergoing a temporary VAD placement (52). Such a technique can be employed in larger children with sufficient aortic valve annulus size, and can likely be implanted in the same amount of time as a prosthetic valve. Transcatheter or open rapid-deployed aortic valve prosthesis are also other options that that have been described in adults and can be conceivably used in older, adult-sized children (53).

Another strategy to eliminate aortic valve insufficiency in pediatric VAD patients is complete valve closure. In this technique, if the leaflets are durable enough to hold sutures, then suturing them together can be more expedient than replacement. In infants with especially thin aortic leaflets, valve closure can be accomplished by excising the leaflets and suturing a patch to the annulus to accomplish a partition between the left ventricle aorta (54). An important consideration in aortic valve closure is that all of the cardiac output is delivered by the VAD and thus any significant device malfunction or inflow/outflow obstruction would have immediately dire consequences. Nonetheless, the closure technique is especially useful in the infant and small child where no traditional prosthetic valve options exist. Percutaneous aortic valve closure has also been reported in adults using an Amplatzer device (Abbott Cardiovascular, Plymouth, MN, USA) as a rescue strategy for a very large regurgitant valve after VAD implantation (55). In patients with a Damus-Kaye-Stansel connection with neoaortic regurgitation, external ligation of the neoaorta has also been described (56).

### 5.3. Tricuspid and pulmonary valve disease

Tricuspid regurgitation can be the result of primary annular dilation as well as leaflet and subvalvar apparatus abnormalities. In the setting of a biventricular circulation with a VAD supporting a failing, systemic left ventricle, a leftward septal shift can induce tricuspid regurgitation, which can be prevented by titrating VAD speed gradually under echocardiographic monitoring of septal position and tricuspid valve function (57). Tricuspid regurgitation can also be the result of a dysfunctional, dilated, or pressure-overloaded right ventricle. In the latter setting, tricuspid regurgitation generally improves with the placement of a systemic VAD (58), possibly because of the reduced the reduced right ventricle afterload.

However, significant tricuspid regurgitation that persists or develops after LVAD implantation in adult patients is associated with increased mortality (58, 59). One can speculate that the hemodynamically significant persistent or *de novo* tricuspid regurgitation that is associated with increased mortality after LVAD implantation is a surrogate marker of right ventricle dysfunction and poor right heart cardiac output. Tricuspid valve repair may be beneficial in instances where there is primary morphological abnormalities of the valve and adequate or recoverable right ventricle function. However, it is not as evident if tricuspid valve repair in the setting of functional tricuspid regurgitation and a dysfunctional right ventricle would provide any benefit. Further, it has been reported that tricuspid valve repair for at least moderate tricuspid regurgitation does not confer any survival benefit after LVAD implantation in adults, possibly due to a significant recurrence of tricuspid regurgitation (59). Data regarding the impact of *de novo* or preexisting tricuspid regurgitation on outcomes in pediatric VAD patients are lacking. Nonetheless, most pediatric heart surgeons are familiar with tricuspid valve repair techniques, and thus we feel that it is reasonable to repair an insufficient tricuspid valve in pediatric patients with depressed right ventricle function. In smaller children, a suture annuloplasty at the commissures is usually sufficient, while in larger children, an annuloplasty ring can be implanted.

Pulmonary valve insufficiency can occur in pediatric patients with congenital heart disease who had prior transannular patch or right ventricle to pulmonary artery conduit placement. These patients would need replacement with a valved conduit or bioprosthetic valve in the setting of a right-sided VAD placement should there be significant native or conduit valve insufficiency.

## 6. Case example

**Figure 2:**
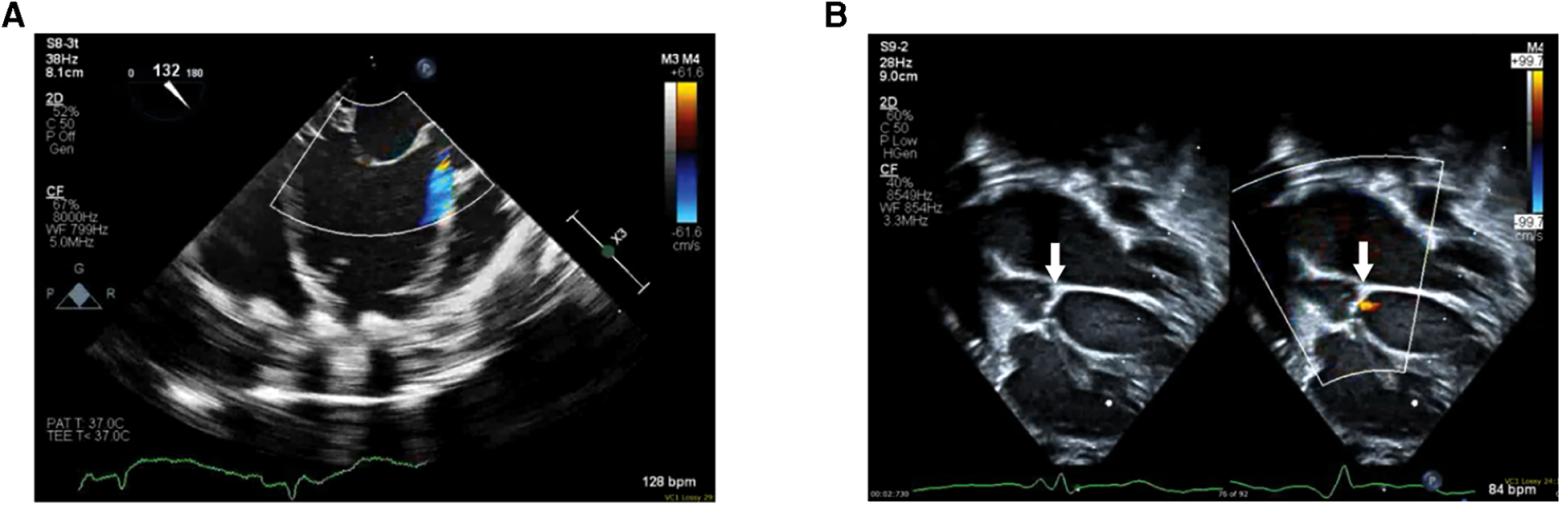
(**A**) Pre-VAD implantation echocardiogram with color doppler mapping demonstrating significant AI. (**B**) Post-VAD implantation 2D echocardiogram (left) with color-doppler comparison (right) demonstrating the patch placed at the ventriculoaortic junction (white arrows) and trivial patch margin leak.

The patient was a 9-day-old, 2.7 kg female born at full term who presented with tachypnea, retractions, and cyanosis. An echocardiogram in clinic was notable for poor left ventricle function and she was immediately admitted to the intensive care unit. She was intubated for respiratory distress and started on empiric antibiotics. She was then transferred to our center for further care. Echocardiogram upon transfer demonstrated severely reduced left ventricle systolic function, mildly depressed right ventricle systolic function, and no semilunar or atrioventricular valve insufficiency. She was then evaluated and listed for heart transplantation. Five days after admission, the patient developed hemodynamic instability despite increased inotrope infusions and thus underwent urgent cannulation for venoarterial extracorporeal membrane oxygenation followed by balloon atrial septostomy for additional decompression of the left heart. She was then transitioned to a Berlin Heart EXCOR VAD two weeks later. Echocardiogram prior to VAD implantation demonstrated the presence of a nonrestrictive atrial septal defect with left to right shunting, severely depressed left ventricle systolic function, left ventricle dilation, mild mitral insufficiency, moderate and continuous AI (Figure 2A), mildly reduced right ventricle function, trivial tricuspid insufficiency, and mild to moderate mitral insufficiency. Given the degree of aortic insufficiency, an aortic valve repair was also undertaken in addition to the atrial septal defect closure and VAD placement. Via median sternotomy, cardiopulmonary bypass was accomplished with ascending aorta and bicaval cannulation. A 5 mm inflow cannula was inserted through the left ventricle apex. Cardioplegic arrest was then accomplished by administering Del Nido solution into the aortic root. A longitudinal aortotomy was then performed on the anterior ascending aorta and extended into the noncoronary sinus to expose the aortic valve. The leaflets were very thin and were noted not to coapt centrally. Because the leaflets were too fragile to hold a central coaptation stitch, we elected to close the ventriculoaortic junction with a patch of bovine pericardium. The leaflets were excised and the bovine pericardial patch was sewn to the annulus with a continuous 7-0 polypropylene suture. The aortotomy in the noncoronary sinus region was then closed, and a slightly beveled 8 mm vascular graft was anastomosed to the anterior aortotomy. The vascular graft was then connected to a 5 mm Berlin outflow cannula that had been previously tunneled through the body wall. The atrial septal defect was closed with a Gore-Tex patch. The VAD cannulae were connected to a 10 ml Berlin Heart Excor blood pump. The heart and VAD were deaired and the aortic cross-clamp was removed. The patient was weaned off cardiopulmonary bypass with the level of VAD support titrated while using echocardiogram to determine ventricular septal position and the degree of tricuspid regurgitation. Total cardiopulmonary bypass time was 169 min, with an aortic cross-clamp of 75 min. Transesophageal and epicardial echocardiogram demonstrated a trivial ventriculoaortic patch margin leak (Figure 2B), no residual atrial level shunting, good right ventricle function, and laminar flow into the inflow cannula. The patient recovered uneventfully and then underwent orthotopic heart transplantation two months after VAD implantation. The patient required the placement of a gastric tube for feeding difficulties and was discharged 6 weeks after heart transplantation.

## 7. Summary

Durable VADs have improved the outcomes of children with end-stage heart failure. The efficacy of VAD therapy in children is affected by valvular heart disease. Clinicians treating pediatric VAD patients need to understand how valvular heart disease can impact the efficacy of VAD support in the varying scenarios of cannulation arrangement, type and location of the valvular heart disease, and type of device. Knowledge of the different surgical techniques to address an insufficient semilunar or atrioventricular valve in the pediatric VAD patient is also important in maximizing the efficacy of VAD therapy while minimizing the risk of surgery and preserving ventricular function. Much of the evidence guiding the current management of pediatric VAD patients with valvular heart disease is taken from the published experience in adult VAD patients and therefore future investigation should specifically study this area to provide pediatric-relevant practice guidelines.
